# Living in the Country

**Published:** 1857-03

**Authors:** 


### LIVING IN THE COUNTRY

AND doing business in town, is a "dog's life," from beginning to end, as far as New York is concerned. Instead of adding to one's comfort and quiet, it diminishes both. So far from promoting health, it undermines it; while in a business point of view, it is attended with a multitude of annoyances of every variety. We have tried it under very favorable circumstances, and speak from experience. We know that many persons think that they would like nothing better than to be able to work in town and live in the country. In some few cases it may be a comfort; it is when a man can afford to go to his place of business not sooner than ten in the morning; or if he does not go at all for any day, or two or three of any week in the year, it makes no kind of difference, having persons on the spot who will do just as well. But to be the main spoke in the wheel of any establishment, whose punctual and daily presence is indispensable, it is an unmistakable bore to live out of the city limits.

The semi-citizen is in a hurry from one year's end to another. When he goes to bed at night, among his last thoughts are—and there is an anxiety about it—that he may oversleep himself, or that the cook may be behind time with his breakfast; so going to sleep with these thoughts, the instant he wakes in the morning there is a start, and the hurry begins—he opens his eyes in a hurry, to determine by the quality of the light whether he is in time. His toilet is completed with dispatch, but instead of composedly waiting for breakfast-call, his mind, even if not on his business, will be in the kitchen. Can a man converse composedly with his family, when the fear is uppermost of his being left by the train? It is impracticable. Even with the case in a thousand, where the cook is a minute-man, he can't for the life of hime at with a feeling of leisure: may be his watch is a little slow; may be the train is a little before time, and the result is, a hurried and unsatisfactory meal, to say the least of it, under the most favorable circumstances; but suppose the cook is like the multitude of her class—never before, but always behind the time—what a fretting feeling is present, mad as fire, yet afraid to say anything; soon the wife gets the contagion, and then the play begins; stand about everybody.

You are deposited in the cars for town; accidents and delays will occur; your mind is in your office, may be a customer is waiting, or you are pressed for time to meet an engagement. As soon as mid-day is past, the solicitude begins lest circumstances should prevent your departure by a specified train; this increases as the hour draws near, and when we take into account the dilatory nature of most men, it will be a marvel if some one is not late in meeting you, or making an expected payment; or a customer does not hang on your button-hole, and you don't wish to offend him. In short, there are such a multitude of causes in operation to crowd the last moments of the business day, that we do not believe that one semi-citizen in a hundred, of any day, walks to the depot from his place of business with a feeling of quiet leisure. When you get home, you are too tired and too hungry to be agreeable until you get your last meal; even then there is a calculation about getting to bed early, so as to have your full sleep by morning. We ask, where is the "quietude" of a life like this? It does not exist. Such a man is an entire stranger to composure of mind. One beautiful morning a sprightly young gentleman entered the cars just as they were moving off. We had seen him often, always in a hurry, always in a pleasant humor. He said to a friend, as he took his seat: "I've been in a hurry from morning until night for the last two years—always on the stretch, but never left. Came very near it this time." Soon afterwards it appeared that he had been industriously engaged the whole of that time, and had accomplished a great deal; for he had, in various directions, disposed of seventy thousand dollars belonging to a public institution, of which he was the custodian. If this incessant hurry, from one year's end to another, can promote quietude of mind, can conduce to one's pecuniary advantage, can foster domestic enjoyments, it is new to us. We think, rather, that it tends to fix on the mind a stereotype impression of anxious sadness, which, in the father of any family, to be seen every day, must have a decided effect in subduing that spontaneous joyousness which should pervade the countenance of every member of a happy household.

There is one little matter which we prefer to speak of before dismissing the subject, which we consider of vital importance, and is the idea which led to the penning of this article:

A daily action of the bowels is essential to good health under all circumstances; the want of it engenders the most painful and fatal diseases. Nature prompts this action with great regularity, most generally after breakfast. Hurry or excitement will dispel that prompting, and the result is, nature is baffled. Her regular routine is interfered with, and harm is done. This is a thing which most persons do not hesitate to postpone, and in the case of riding to town, a delay of one or two hours is involved. This never can occur with impunity, in any single instance, to any person living. This very little thing—postponing nature's daily bowel actions—failing to have them with regularity—is the cause of all cases of piles and anal fistulas, to say nothing of various other forms of disease: fever, dyspepsia, headache, and the whole family of neuralgias. A man had better lose a dinner, better sacrifice the earnings of a day, than repress the call of nature; for it will inevitably lead to constipation, the attendant and aggravator of almost every disease. To arrange this thing safely, breakfast should be had at such an early time as will allow of a full half hour's leisure between the close of the meal and the time of leaving for the cars.

